# PTSD Symptoms Are Associated with a Greater Use of Social Camouflaging Strategies in an Eating Disorder Sample with Elevated Autistic Traits

**DOI:** 10.3390/brainsci16030303

**Published:** 2026-03-11

**Authors:** Liliana Dell’Osso, Benedetta Nardi, Martina Calvaruso, Alina Lohse, Cristiana Pronestì, Chiara Bonelli, Gabriele Massimetti, Ivan Mirko Cremone, Mario Luciano, Stefano Pini, Andrea Fiorillo, Barbara Carpita

**Affiliations:** 1 Department of Clinical and Experimental Medicine, University of Pisa, 56126 Pisa, Italyalinalohse@me.com (A.L.); cristianapronesti@gmail.com (C.P.);; 2 Department of Psychiatry, University of Campania “L. Vanvitelli”, 80138 Naples, Italy; mario.luciano@unicampania.it (M.L.);

**Keywords:** eating disorder, trauma, symptomatologic PTSD, trauma- and stress-related symptoms, autism spectrum, autistic traits, camouflaging, social camouflaging

## Abstract

**Background**: Eating disorders (EDs) frequently co-occur with trauma-related symptoms and elevated autistic traits (ATs), both of which contribute to clinical complexity. Social camouflaging, strategies used to mask or compensate for ATs, has been increasingly described in ED populations, yet its relationship with trauma-related symptoms remains poorly understood. This study aimed to examine the association between social camouflaging and post-traumatic stress symptoms in individuals with EDs and to evaluate whether trauma-related symptomatology is associated with camouflaging behaviors. **Methods**: A total of 67 ED patients were assessed using the Adult Autism Subthreshold Spectrum, the Trauma and Loss Spectrum—Self Report (TALS-SR), the Camouflaging Autistic Traits questionnaire (CAT-Q), and the Eating Disorder Inventory (EDI-2). Participants were divided into high-trauma-symptoms (HTS) (N = 36, 53.7%) and low-trauma-symptoms (LTS) (N = 31; 46.3%) groups based on TALS-SR criteria. **Results**: The sample was predominantly female (92.5%), and gender distribution differed between groups, which may represent a potential confounding factor and limits the generalizability of the findings. The HTS group reported significantly higher TALS-SR, EDI-2, CAT-Q, and AdAS Spectrum scores, although for the latter the *p*-value was barely significant (*p* = 0.046). No differences emerged in the distribution of ED diagnoses between groups. CAT-Q scores were significantly positively correlated with TALS-SR total scores and with domains related to reaction to losses, maladaptive coping, avoidance/numbing, and personal vulnerability. Regression analyses showed that overall trauma-related symptoms were significantly associated with greater camouflaging; however, the proportion of explained variance was modest, suggesting that trauma-related symptoms represent only one of multiple factors linked to camouflaging. **Conclusions**: Among individuals with EDs, higher trauma-related symptomatology is linked to greater use of social camouflaging strategies. These findings suggest that camouflaging may represent a transdiagnostic correlate connecting neurodevelopmental vulnerability and trauma responses within ED populations, underscoring the importance of integrated assessment and trauma-informed care.

## 1. Introduction

Eating disorders (EDs) are severe psychiatric conditions characterized by persistent disturbances in eating behaviors, body image, and emotion regulation, and they are frequently associated with significant functional impairment and psychiatric comorbidity [1]. According to the most recent edition of the Diagnostic and Statistical Manual for Mental Disorder (DSM-5-TR), the category of EDs encompasses several diagnoses such as anorexia nervosa (AN), bulimia nervosa (BN), and binge eating disorder (BED). These disorders disproportionately affect females and represent a significant public health concern, with estimated lifetime prevalence of 8.4% among women and 2.2% among men [2]. A growing body of evidence indicates that EDs are not isolated syndromes but rather emerge from the interaction of biological, psychological, and interpersonal vulnerabilities, often shaped by early adverse experiences and neurodevelopmental features [3]. Among these vulnerabilities, two psychopathological dimensions have received increasing attention: exposure to traumatic events and presence of autistic traits (ATs). Both factors appear to contribute to the onset, maintenance, and clinical complexity of EDs, suggesting the presence of shared transdiagnostic mechanisms that remain insufficiently understood.

Traumatic experiences, including emotional, physical, and sexual abuse, neglect, interpersonal victimization, and exposure to highly stressful or life-threatening events, have been described as disproportionately common among individuals with EDs [4], and in particular, in people with AN [5]. Notably, the prevalence of post-traumatic stress disorder (PTSD) or clinically significant trauma-related symptoms among individuals with EDs is markedly elevated. Recent epidemiological and clinical studies report PTSD rates ranging from 25% to over 50% in ED populations, depending on diagnostic subtype and assessment method [6,7,8]. Multiple studies have highlighted a consistent association between various forms of childhood maltreatment and the later development of EDs [9,10,11]. In light of this, many authors have hypothesized that early traumas may act as environmental risk factors for ED development, potentially through mechanisms such as altered interoception or feelings of ineffectiveness, impulsivity, and interpersonal distrust [3,4,9,12]. Interpersonal trauma, in particular, appears to exert a potent influence on the development of restrictive, binge–purging, and dysregulated eating behaviors [13,14,15,16]. On the other hand, some authors suggested heightened vulnerability to trauma-related symptoms in people with EDs due to atypical amygdala functioning, leading to altered processing of negative emotional and body-related stimuli [17] and hypothalamic–pituitary–adrenal-axis (HPA-axis) hyperactivation [18].

Parallel to research on trauma, a growing body of work has examined the role of ATs in EDs, and particularly in AN. Indeed, ATs appear to be overrepresented in ED populations, with evidence supporting both categorical diagnostic overlap and dimensional comorbidity [19,20]. These features may contribute to both the emergence and maintenance of ED symptoms [20,21]. In this framework, many authors have proposed a potential reconceptualization of some forms of EDs as female phenotypes of the autism spectrum disorder (ASD), due to similarities in rigidity, sensory atypicality, social cognitive difficulties, alexithymia, narrow interests and cognitive inflexibility, in which autistic features may be overshadowed or masked by social camouflaging strategies learned as compensatory behaviors, often represented in autistic females [22]. Social camouflaging refers to strategies used for masking, compensating for, or hiding ATs in social situations [23]. These behaviors include imitating facial expressions, forcing eye contact, or avoiding discussions about personal interests. Although initially described in ASD individuals, particularly females [24,25], camouflaging behaviors are increasingly recognized in individuals with subclinical ATs, and in other psychiatric conditions associated with higher ATs, such as EDs or borderline personality disorder [26,27]. While camouflaging may facilitate short-term social adaptation, it is consistently associated with heightened emotional exhaustion, anxiety, depressive symptoms, suicidality, and decreased well-being [28,29,30,31]. Autistic women in particular show increased risk of suicidal thoughts and behaviors that can impair overall functioning and quality of life [32]. It should be noted that social camouflaging has also been linked to disordered eating behaviors: in particular, social camouflaging strategies predict greater ED symptom severity in both ASD adults [33] and non-clinical samples [27]. Similar associations have been reported in female patients with borderline personality disorder, where camouflaging seems to predict ED symptomatology [26]. These findings suggest that camouflaging may function as a maladaptive coping strategy that influences ED processes, particularly in individuals with neurodevelopmental vulnerabilities and heightened sensitivity to interpersonal threat.

Recent findings also indicate that ATs may increase the vulnerability towards traumatic experiences. Individuals with elevated ATs are more likely to experience interpersonal victimization, coercion, and abuse and to develop PTSD-like symptoms following trauma [34,35,36,37,38,39]. Mechanisms proposed to explain this increased vulnerability include social naïveté, difficulties interpreting others’ intentions, difficulties in adjusting to change, sensory overwhelm, and tendencies toward rumination, features that may amplify the impact of traumatic events. Accordingly, traumatic exposure in individuals with elevated ATs may give rise to complex trajectories in which neurodevelopmental and trauma-related vulnerabilities reinforce one another and contribute to more severe or atypical ED presentations [40].

Interestingly, some authors hypothesized that traumatic experiences, especially of an interpersonal nature, may exacerbate or shape the development of camouflaging strategies. Individuals with ATs who have experienced interpersonal trauma may engage more intensely in masking behaviors as a means of preventing further victimization, managing social threat, or conforming to perceived norms in invalidating environments [29,30].

Despite the independent associations reported between post-traumatic symptoms, ATs, and ED psychopathology, few studies have examined how trauma-related symptoms might be associated with the use of camouflaging strategies in individuals with EDs. Trauma-related reactions, such as avoidance, emotional numbing, hyperarousal, maladaptive coping, and disrupted self-concept, may plausibly increase reliance on camouflaging as a means of managing social environments perceived as threatening. Conversely, persistent camouflaging may itself increase susceptibility to internalizing symptoms and amplify trauma-related stress responses. Given the high prevalence of both trauma exposure and ATs in ED populations, camouflaging may represent a key, yet understudied, transdiagnostic mechanism linking neuroatypicality, traumatic experiences, and clinical presentation of EDs.

Within a dimensional and transdiagnostic framework, EDs can be conceptualized as the outcome of interacting neurodevelopmental vulnerabilities and adverse environmental experiences [19,20]. Individuals with elevated ATs often present difficulties in social cognition, emotion regulation, sensory processing, and cognitive flexibility, which may increase exposure to interpersonal stressors and victimization and heighten vulnerability to trauma-related symptoms [34,35,36,37,38,39,40]. In turn, trauma-related reactions, such as avoidance, emotional numbing, hyperarousal, maladaptive coping, and disrupted self-concept, are highly prevalent in ED populations and are associated with greater symptom severity and interpersonal dysfunction [6,7,8,13,14,15,16]. Within this context, social camouflaging may emerge as a coping response aimed at reducing perceived social threat, preventing further victimization, and facilitating social adaptation in invalidating or unsafe environments. While camouflaging may confer short-term interpersonal benefits, its persistent use has been associated with increased psychological distress, emotional exhaustion, internalizing symptoms, suicidality, and poorer well-being [28,29,30,31,32]. Moreover, growing evidence suggests that camouflaging is linked to greater ED symptomatology in individuals with autism spectrum conditions, subthreshold ATs, and related clinical populations [26,27,33]. Taken together, these findings support a conceptual model in which social camouflaging represents a maladaptive coping strategy that may bridge ATs and trauma-related symptomatology within ED populations.

Based on the conceptual framework outlined above, the present study was designed to test the following hypotheses. First, we hypothesized that patients with EDs and high trauma symptoms (HTS) would report significantly higher levels of social camouflaging and ED severity compared to ED patients with low trauma symptoms (LTS). Second, we hypothesized that, across the entire ED sample, trauma-related symptom severity would be positively correlated with the extent of social camouflaging. Third, we hypothesized that trauma-related symptoms would be significantly associated with the use of social camouflaging strategies.

## 2. Materials and Methods

### 2.1. Study Sample and Procedures

The study included 67 ED patients enrolled at the Psychiatric Department of the Azienda Ospedaliera Universitaria Pisana (AOUP). Among them, 36 subjects also met criteria for significant trauma- and stress-related symptoms according to TALS-SR and were included in the HTS group. Participants were aged between 18 and 70 years. Exclusion criteria were: age under 18, significant language or intellectual impairments, schizophrenia, substance abuse history, major neurological or medical conditions, or inability to provide written informed consent or complete psychometric assessments independently. All participants were assessed by trained psychiatrists, with diagnoses made according to DSM-5-TR criteria. Detailed information about the study was provided, and participants were given the opportunity to ask questions before signing written informed consent. The study was conducted in accordance with the Declaration of Helsinki and approved by the AOUP Ethics Committee.

The sample size was determined by the number of eligible patients consecutively assessed during the study period who met the inclusion criteria and provided complete data on the relevant psychometric measures. Performing a power analysis, we found for our sample a power of 1.000 on TALS-SR total score, 0.748 on CAT-Q total score and 0.898 on CAT-Q total score.

All participants were assessed with the Adult Autism Subthreshold Spectrum (AdAS Spectrum) for the investigation of autistic features, the Trauma and Loss Spectrum—Self Report (TALS-SR) for the assessment of trauma- and stress-related symptoms, the Camouflaging Autistic Traits Questionnaire (CAT-Q) for the evaluation of social camouflaging strategies, and the Eating Disorder Inventory (EDI-2) for the investigation of altered eating behaviors.

Participants were recruited based on a diagnosis of an eating disorder; elevated ATs were not an inclusion criterion but were assessed dimensionally and emerged as a characteristic of the sample.

The study was conducted from 2023 to 2024.

### 2.2. Measures

#### 2.2.1. The AdAS Spectrum

The AdAS Spectrum is a 160-item self-assessment tool composed of yes/no questions, grouped into seven distinct domains. It is designed to evaluate a wide range of autism-related characteristics and symptoms in adults without cognitive or language impairments. The seven domains include aspects related to *childhood and adolescence*, *verbal* and *non-verbal communication*, *empathy*, *inflexibility and adherence to routine*, *restricted interests and rumination*, and *hyper- or hyporeactivity to sensory stimuli*. Scores range from 0 to 160, with higher scores indicating greater symptomatology. Validation studies have demonstrated that the instrument has high internal consistency, strong test–retest reliability, and good convergent validity when compared with other autism spectrum measures [41].

#### 2.2.2. The CAT-Q

The CAT-Q is a self-administered questionnaire developed to assess camouflaging behaviors associated with autism spectrum traits in both clinical and general populations. It includes 25 items divided into three subscales, *Assimilation*, *Masking*, and *Compensation*, rated on a seven-point Likert scale [42]. Scores range from 25 to 175, with higher scores indicating greater symptomatology. The Italian version of the CAT-Q exhibited excellent psychometric properties, such as internal consistency (Cronbach’s alpha = 0.904), and robust test–retest reliability (all the ICC values for CAT-Q factors and total score were above 0.90), alongside strong positive correlations with the AdAS Spectrum (Pearson’s r correlation = 0.652) [43]. These findings support the CAT-Q as a reliable and valid tool for the assessment of social camouflaging behaviors [43].

#### 2.2.3. The TALS-SR

The TALS-SR is a self-report measure designed to assess lifetime exposure to a range of traumatic and loss-related experiences, along with associated stress symptoms, behaviors, and individual traits that may increase vulnerability to stress-related disorders. It comprises 116 yes/no items organized into nine categories: *Loss Events*, *Grief Responses*, *Potentially Traumatic Events*, *Reactions to Losses or Upsetting Events*, *Re-experiencing*, *Avoidance and Numbing*, *Arousal*, *Maladaptive Coping*, and *Personal Characteristics/Risk Factors*. Elevated scores reflect more severe symptom expression. Scores range from 0 to 116, with higher scores indicating greater symptomatology. The tool has shown robust psychometric validity and reliability overall [44]. In line with the previous literature, in this work, the TALS was also employed for assessing the presence of full-blown PTSD symptoms according to DSM-5 criteria, by using an algorithm based on TALS-SR items that match DSM-5 symptomatologic criteria for PTSD [45]. The use of a TALS-SR-based algorithm to identify symptomatologic PTSD is consistent with a dimensional approach to trauma-related psychopathology and has been widely adopted in both clinical and research settings. Compared with structured clinical interviews, self-report spectrum instruments allow for the assessment of a broader range of subthreshold and atypical trauma-related symptoms, which may be particularly relevant in vulnerable populations such as individuals with elevated ATs and EDs, who may experience difficulties in recognizing, verbalizing, or contextualizing traumatic experiences [34,35,46,47,48,49,50]. Accordingly, in the present study the HTS classification should be interpreted as reflecting the presence of clinically significant trauma-related symptomatology, without considering the presence of a high-magnitude traumatic experience as described in DSM-5 criterion A. This approach is particularly useful in our population, considering that subjects with ATs may be more vulnerable to life events and eventually develop PTSD-like symptoms even after minor traumatic experiences [34,35,36,37,38,39].

#### 2.2.4. The EDI-2

The EDI-2, developed by Garner et al. in 1991, is a self-administered questionnaire consisting of 91 items divided into 11 subscales and was designed to assess psychological traits and symptomatology associated with EDs, including AN, BN and BED [51]. These subscales include *Drive for Thinness*, *Bulimia*, *Body Dissatisfaction*, *Ineffectiveness*, *Perfectionism*, *Interpersonal Distrust*, *Interoceptive Awareness*, *Maturity Fears*, *Asceticism*, *Impulsivity*, and *Social Insecurity*. The initial eight subscales were preserved from the original EDI framework, whereas the additional three subscales were incorporated to enhance the instrument’s capability to delineate the complex psychological dimensions pertinent to ED psychopathology [51]. Responses are rated on a 6-point Likert scale. Scores range from 0 to 273, with higher scores indicating greater symptomatology. The broad scope of these subscales allows for a comprehensive evaluation of both the symptomatic and psychological dimensions of EDs.

### 2.3. Statistical Analysis

Sociodemographic characteristics were compared between groups using independent-samples *t*-tests and Chi-square tests.

An independent-samples *t*-test was also used for comparing mean scores on the AdAS Spectrum, CAT-Q, EDI-2, and TALS-SR questionnaires between patients with HTS and LTS.

Chi-square analyses were then conducted to examine differences in the representation of specific ED diagnoses—including AN restrictive (AN-R), AN binge–purging (AN-BP), BN, and BED—between the two groups.

Subsequently, Pearson’s correlation analyses were performed to evaluate potential associations between CAT-Q and TALS-SR scores.

Finally, two linear regression analyses were conducted to investigate whether trauma-related symptoms were significantly associated with the use of camouflaging strategies. In the first model, the TALS-SR total score was used as the independent variable; in the second model, TALS-SR domain scores were entered as the independent variables. In both models, the CAT-Q total score was included as the dependent variable.

Age and gender were not included as covariates in the regression models. This choice was guided by the exploratory nature of the study and the relatively limited sample size, which constrained the number of variables that could be reliably included without increasing the risk of overfitting and unstable parameter estimates. In addition, the primary aim of the regression analyses was to examine the association between trauma-related symptomatology and social camouflaging within the overall ED sample, rather than to model demographic influences on camouflaging.

Correlation and regression analyses were conducted in the overall sample of individuals with EDs in order to examine the dimensional association between trauma-related symptom severity and social camouflaging strategies, independent of categorical group assignment. This approach is consistent with a spectrum-based and transdiagnostic framework, in which trauma-related symptoms are conceptualized as continuously distributed, and allows for the investigation of whether increasing trauma burden is associated with greater camouflaging across the full range of symptom expression.

## 3. Results

The sample was composed of 5 males (7.5%) and 62 females (92.5%). A total of 36 participants (53.7%) fulfilled the symptomatologic criteria for clinically relevant trauma- and stress-related symptoms according to TALS-SR, hereafter referred to as the HTS group.

Significant sociodemographic differences were observed between the HTS and LTS groups. Specifically, HTS patients were significantly younger (mean age: 30.61 ± 11.79 vs. mean age: 38.42 ± 15.40, t = 2.308, *p* = 0.025) and included a higher proportion of females (F = 36, 100% vs. F = 26, 83.9%, X^2^ = 6.275, *p* = 0.012) compared to LTS patients.

In the HTS group, the most frequently reported traumatic or sub-traumatic experience (according to items 38–54 of the TALS-SR spectrum) was being exposed to unwanted sexual advances, while in the LTS group it was repeated severe arguments in the family (see Table 1).

Results from the *t*-test analysis showed that participants in the HTS group reported significantly higher total scores on both the TALS-SR and CAT-Q questionnaires, including on all CAT-Q domains, on specific EDI-2 domains, and on the AdAS Spectrum total score, although for the latter the *p*-value was barely significant (*p* = 0.046) (see Table 2).

A subsequent Chi-square analysis revealed no significant differences in the distribution of specific ED diagnoses—AN-R (LTS: N = 5, 16.1%; HTS: N = 8, 22%), AN-BP (LTS: N = 3, 9.7%; HTS: N = 4, 11.1%), BN (LTS: N = 12, 38.7%; HTS: N = 19, 52.8%), and BED (LTS: N = 11, 35.5%; HTS: N = 5, 13.9%)—between patients with and without comorbid HTSs (X^2^ = 4.317, *p* = *0*.229).

Moreover, results from the Pearson correlation analysis showed that CAT-Q total scores were significantly and positively correlated with TALS-SR total scores. (see Table 3).

Results from the linear regression analysis performed using the CAT-Q total score as the dependent variable and the TALS-SR total score as the independent variable highlighted how trauma-related symptoms were statistically associated with a higher use of camouflaging strategies (see Table 4).

Lastly, results from the second linear regression analysis, performed with the CAT-Q total score as the dependent variable and TALS-SR domain scores as independent variables, indicated that the TALS-SR *Maladaptive Coping* domain was significantly and positively associated with the use of camouflaging strategies, whereas the *Loss Events* domain was negatively associated with these behaviors (see Table 5).

## 4. Discussion

The aim of this study was to explore the interplay between social camouflaging and the presence of trauma-related symptoms in individuals with EDs, a population associated with the presence of high ATs.

First, our study highlighted a consistent presence of HTS in our sample (53.7%), stressing the already known association between trauma- and stress-related symptoms and EDs [5,6,7,8,40,52,53,54]. Noticeably, according to our data, the most frequently reported traumatic or sub-traumatic event in the HTS group was being exposed to unwanted sexual advances. In addition, the other more frequently reported (sub)traumatic experiences were of an interpersonal nature. This is in line with the previous literature, which stressed an increased presence of interpersonal traumatic experiences in patients with EDs [3,4,5,8,9,11,13,14,15,16,55,56]. It should also be mentioned that, while in ED populations a higher presence of ATs is reported [19,20,40,57,58], the latter are considered a risk factor for exposure to interpersonal trauma [34,35,36,37,38,39].

Although our sample consisted mostly of females, reflecting the well-known higher prevalence of EDs in this gender, our data appear to confirm the association previously reported in the literature between PTSD and being female [59,60,61]. In this framework it should be noted that, although the literature reports higher rates of PTSD–EDs comorbidity in females, this finding may be confounded by the systematic underrepresentation and limited investigation of male populations in this field [62]. Noticeably, in a large-scale study including a substantial sample of males (n = 2382) and females (n = 3310), interpersonal forms of trauma, PTSD, and subthreshold/partial PTSD were reported to be significantly higher in both male and female individuals diagnosed with EDs [62].

In addition, our data also reported a significantly younger age in patients who belonged to the HTS group; this finding could be explained by hypothesizing that patients with EDs and comorbid trauma- and stress-related symptoms may eventually seek treatment at an earlier age than those without, due to a globally more severe clinical picture. Studies in this field indicate that EDs and trauma symptomatology comorbidity is associated with greater clinical severity, earlier traumatic exposure, and a younger age at onset of ED symptoms [7,10,63,64,65,66,67,68,69], which may indirectly support the hypothesis that trauma-related factors could contribute to an earlier manifestation and potentially to earlier help-seeking behaviors in this population.

As expected, the HTS group reported significantly higher TALS-SR total score, further confirming the higher severity of trauma-related symptomatology in this group and in the AdAS Spectrum total score, although the *p*-value barely reached significance. In addition, the HTS group obtained significantly higher scores on the EDI-2 total score. This finding is consistent with the literature stressing a link between ED symptoms and trauma- and stress-related symptomatology [69]. Traumatic experiences are commonly found in the clinical history of individuals with EDs [5,8,40,52,67]. Some studies reported that early interpersonal trauma plays a key role as an environmental risk factor in the development of EDs among vulnerable subjects [4,8]. Moreover, recent research has investigated possible neurobiological shared mechanisms of EDs and symptomatologic PTSD [54]. On the other hand, the increased vulnerability of ED subjects towards the development of trauma-related symptoms is further supported by studies on neural processes and neuroimaging findings [17,18].

Moreover, our findings indicating that patients with HTS obtained significantly higher scores in the EDI-2 domains of *Ineffectiveness*, *Interoceptive Awareness*, *Impulsivity*, and *Social Insecurity* are consistent with the previous literature. Indeed, previous research highlighted the correlation between altered interoceptive awareness and feelings of ineffectiveness in the relationship between traumatic events and the development of EDs [9,69]. Furthermore, in a network analysis exploring the relationship between EDs and trauma- and stress-related symptoms, ineffectiveness exhibited, as a symptom, the highest centrality, followed by interoceptive awareness [65]. Considering the EDI-2 Impulsivity domain, some literature reported how the relationship between early traumatic events—particularly emotional and sexual abuse during childhood—and subsequent development and maintenance of EDs may be correlated with anger, impulsivity, and compulsivity [13,14,15,16,69]. These psychopathological features may lead to deficits in emotion regulation and/or behavioral control, as well as to maladaptive coping strategies for managing negative emotional states and trauma- and stress-related symptoms [13,14,15,16]. Finally, the higher scores in the *Social Insecurity* domain of EDI-2 observed in the HTS group of our sample are also consistent with previous studies, which found that patients with EDs and post-traumatic symptoms exhibit higher levels of insecurity and interpersonal distrust [69]. On the other hand, research has shown that experiences of marginalization and various forms of discrimination, such as racism, weight stigma, and sexism, may compound the risk of developing EDs and predict body image dissatisfaction and disordered eating behaviors [56].

Our results revealed no significant differences in the distribution of specific ED diagnoses (AN-R, AN-BP, BN, and BED) between the HTS and LTS groups, consistent with the previous literature [69,70], although limited power may have reduced the ability to detect diagnostic distribution effects.

According to our data, both groups reported AdAS Spectrum mean scores above the cut off for significant ATs (43 points), with a higher score in the HTS group [71]. The presence of high AdAS Spectrum scores in both groups, considering that the sample was composed of ED patients, is in line with the increasing literature about the link between EDs and the autism spectrum [19]. Indeed, a substantial body of literature has consistently documented strong links between ATs and EDs. Previous studies have reported familial aggregation between autism spectrum conditions and EDs, overlapping symptom profiles, particularly in domains such as cognitive rigidity, social communication difficulties, and emotional regulation, and high rates of comorbidity. On this basis, several authors have proposed that, in a subgroup of patients, EDs may represent a gender-specific phenotypic expression of the autism spectrum, characterized by socially mediated symptom presentations and compensatory behaviors [12,19,20,21]. In the scientific literature, trauma- and stress-related symptoms have been associated with greater ATs [24,36,40,43,47,72,73,74]: the present study globally confirmed this data, although it is possible that the difference in our sample was only barely significant (*p* = 0.46) due to the overall presence of high Ats.

Noticeably, the HTS group reported significantly higher scores in the CAT-Q total and subscale scores than the LTS group. Moreover, in the whole sample, significant positive correlations were found between trauma-related symptomatology (TALS-SR total scores) and social camouflaging (CAT-Q total scores). These data are further confirmed by the linear regression analysis, which highlighted that total TALS-SR scores are significantly and positively associated with CAT-Q scores.

This finding is especially relevant considering that the sample was composed exclusively of individuals with EDs. It suggests that, within this clinical sample, the presence of significant trauma-related symptoms may be associated with greater social camouflaging strategies. Social camouflaging is well documented among individuals on the autism spectrum [23], particularly among females [24,25,75], follows different developmental trajectories [76,77] during life, can vary in manifestation due to cultural variation [78] and, while it may be grant a better adjustment to social environment, it has been associated with increased psychological distress, emotional exhaustion, poorer well-being, and a heightened risk of internalizing disorders in the long term [28,29,30,31,32,79]. While, as previously described, increasing literature is describing the presence of undiagnosed ASD or subclinical ATs among subjects with EDs [20,21,40,80], recent studies also reported a role of social camouflaging in the relationship between ATs and EDs. For instance, Bradley et al. [33] found that high levels of camouflaging behaviors significantly predicted ED symptoms in ASD adults. Similarly, Carpita et al. [27] reported that, in female university students, social camouflaging served as a significant correlating factor between ATs and orthorexic tendencies. Moreover, recent findings indicated that the use of social camouflaging strategies was higher and closely associated with ED symptoms in BPD patients, a population where both ATs and EDs are frequently reported [26]. From this perspective, while the repeated use of camouflaging strategies may be associated with the development of greater stress-related symptoms as reported in previous studies [28,29,30,31,75,79,81,82,83,84,85], it is possible that, in subjects with ATs, the development of trauma-related symptoms after traumatic experiences (especially if of an interpersonal nature) may be associated with the adoption of camouflaging behaviors as a form of (maladaptive) coping strategy for preventing further adverse relational experiences. This hypothesis aligns with developmental models that link ATs and early adverse experiences to altered neurodevelopmental trajectories and atypical social adaptation strategies [29,48,86,87].

Subsequently, within our entire sample of individuals with EDs, a significant association emerged between social camouflaging and specific dimensions of trauma-related symptomatology. Correlation analyses revealed significant associations between the CAT-Q and TALS-SR total scores. The multiple regression analysis provides a more in-depth understanding: *Maladaptive coping* emerged as the main positive factor associated with the total CAT-Q scores, whereas loss-related events show an inverse relationship with camouflaging. This finding, linking social camouflaging to maladaptive coping strategies typically developed among subjects with symptomatologic PTSD, seems to support the hypothesis that conceptualizes social camouflaging as a possible (maladaptive) coping strategy in response to stressful events of an interpersonal nature, with the aim of managing relational distress among subjects with ATs [29]. However, the cross-sectional design of our study does not allow determination of whether trauma-related symptoms increase camouflaging, whether camouflaging contributes to distress, or whether these processes influence one another bidirectionally.

An additional finding that warrants consideration is the negative association between loss-related events and social camouflaging observed in the regression analysis. At first glance, this result may appear counterintuitive, as exposure to adverse experiences might be expected to increase compensatory social behaviors. One possible interpretation is that experiences of significant loss may reduce individuals’ psychological resources or motivation to sustain effortful camouflaging strategies, leading instead to social withdrawal, disengagement, reduced investment in impression management, or eventually even to attributing more value to seeking deeper connections. It is also possible that loss-related events differ qualitatively from interpersonal trauma in their impact on social functioning. Whereas interpersonal victimization may heighten perceived social threat and promote camouflaging as a defensive coping strategy, loss experiences may elicit grief-related processes that are less directly tied to social conformity or self-monitoring. However, given the relatively small sample size and the exploratory nature of the regression model, this finding should be interpreted with caution and may reflect sample-specific effects or statistical instability. Accordingly, the inverse association between loss events and camouflaging should be considered preliminary and hypothesis-generating, requiring replication in larger and longitudinal studies before firm conclusions can be drawn.

It should be noted that the proportion of variance explained by trauma-related symptomatology was modest (R^2^ = 0.061), underscoring that social camouflaging is a complex and multiply determined behavior and that trauma-related symptoms account for only a limited share of variability in camouflaging behaviors; thus, the observed associations should be interpreted as contributing factors rather than primary determinants.

The decision to examine correlations and statistical relationships in the overall ED sample reflects a dimensional approach to trauma-related psychopathology. While group comparisons highlight clinically meaningful differences between patients with and without symptomatologic PTSD, analyses conducted across the entire sample allow for a more fine-grained evaluation of how trauma-related symptom severity relates to social camouflaging beyond categorical diagnostic thresholds.

This study should be considered in light of several limitations. First, the cross-sectional design precludes any inference about the temporal or causal direction of the relationships among ATs, trauma-related symptoms, social camouflaging, and ED psychopathology. Although trauma-related symptomatology emerged as a significant factor associated with camouflaging behaviors in regression analyses, it is not possible to determine whether trauma-related symptoms increase reliance on camouflaging strategies, whether sustained camouflaging contributes to greater trauma-related distress, or whether both processes reciprocally influence one another over time. Longitudinal studies are needed to clarify these directional pathways. Secondly, PTSD symptomatology was assessed using a self-report spectrum instrument rather than a structured clinical interview. While this approach is well suited to a dimensional evaluation of trauma-related symptoms and may capture clinically relevant subthreshold presentations, it limits direct comparability with categorical interview-based PTSD diagnoses. Another important limitation concerns the exclusive reliance on self-report measures. Trauma-related symptoms and social camouflaging both involve subjective experiences of self-awareness, social monitoring, and emotional distress, which may be differentially perceived or reported by individuals with elevated trauma-related symptomatology. In particular, heightened hypervigilance and self-monitoring associated with trauma exposure may contribute to increased reporting of both trauma symptoms and camouflaging behaviors, potentially inflating observed associations due to shared method variance. The exclusive reliance on self-report instruments may have also introduced reporting biases related to self-awareness, recall, or social desirability, particularly in a population characterized by difficulties in emotional awareness and self-referential processing. Consequently, correlations should be interpreted as reflecting self-perceived psychological processes rather than objective behavioral correspondence. Future studies should adopt multi-method approaches, integrating clinician-administered interviews, behavioral or observational measures, and longitudinal designs to reduce shared method bias and clarify underlying correlation. Third, the relatively limited sample size may have reduced statistical power and may affect the stability and generalizability of regression estimates, particularly in models including multiple variables. As a consequence, the magnitude of the observed effects should be interpreted with caution, and the possibility of over- or underestimation of regression coefficients cannot be excluded. Future studies with larger samples are needed to confirm the robustness of these findings and to allow for more complex multivariate and stratified analyses. In addition, the sample size did not allow us to perform a more in-depth analysis for each specific ED or to evaluate differences depending on exposure to specific traumatic events. Moreover, although significant group differences in age and gender distribution emerged between patients with and without HTS, these variables were not entered as covariates in the regression models. Given existing evidence that both age and gender may influence camouflaging behaviors and trauma-related symptom expression, these demographic differences may have contributed to the observed group effects. Due to the limited sample size and pronounced gender imbalance, it was not methodologically appropriate to conduct covariate-adjusted or stratified analyses. Consequently, as gender was not controlled for in the analyses, it may represent a potential uncontrolled confound when interpreting group differences, and, given the composition of the sample (92.5% female), findings may not generalize to males with EDs. Future studies with larger and more demographically balanced samples are needed to formally disentangle the effects of trauma-related symptomatology from age- and gender-related influences. Also, regression models including multiple trauma-related symptom domains may be susceptible to overfitting, as reflected by the difference between explained and adjusted variance, and should therefore be interpreted cautiously. Lastly, the absence of a non-ED comparison group limits the ability to determine whether the observed associations between trauma-related symptoms and social camouflaging are specific to ED populations or reflect more general transdiagnostic correlations linked to ATs and trauma exposure. Including clinical and non-clinical control groups in future research would help clarify the specificity and generalizability of these findings.

However, despite these limitations, this study highlighted that, in a sample of ED patients with significant ATs, the use of social camouflaging seems to be higher among those with a high prevalence of trauma- and stress-related symptoms, which is statistically associated with the same social camouflaging strategies. The study suggests that, in clinical settings, it is essential to consider social camouflaging as a potential factor linking traumatic history, ED psychopathology and atypical social functioning, such as maladaptive coping. Further research is warranted to elucidate the underlying correlation, developmental pathways, and treatment implications of the relationship between trauma- and stress-related symptoms and social camouflaging in EDs. Integrating trauma-informed approaches with neurodiversity-affirming care may prove particularly beneficial for ED patients; however, additional evidence is needed to support the development of tailored interventions.

## 5. Conclusions

The present study provides preliminary evidence of an association between trauma-related symptom severity and the extent of social camouflaging in individuals with EDs and elevated ATs. These findings support the hypothesis that, in individuals with neurodevelopmental vulnerabilities, traumatic experiences, particularly those that are interpersonal in nature, may reinforce the use of masking and compensatory strategies as a form of maladaptive social adaptation. Recognizing camouflaging as a potential correlator between trauma exposure, ATs, and ED symptomatology may offer new insights into the complex psychopathological trajectories observed in this population. Clinically, these findings highlight the value of integrating trauma-informed and neurodiversity-affirming principles into ED treatment. A neurodiversity-affirming approach may further reduce reliance on exhausting camouflaging strategies by promoting authentic self-expression, accommodating sensory and communication differences, and fostering predictable and safe therapeutic environments. However, trauma-related symptoms showed a statistically significant but modest association with camouflaging. Future research should examine additional factors that may better explain variance in camouflaging behaviors. Accordingly, the findings should be interpreted cautiously, given the cross-sectional design and limited effect size. Future research should examine additional psychological, developmental, and contextual factors that may better account for variability in camouflaging and clarify the directionality of these associations.

## Figures and Tables

**Table 1:** Reported exposure to different traumatic events in the HTS group.

TALS-SR Items (38–54)		HTS Positive Answer N (%)	LTS Positive Answer N (%)
42	Unwanted sexual advances	27 (75.0)	10 (32.3)
39	Repeated severe arguments in your family	26 (72.2)	13 (41.9)
40	Repeatedly teased or harassed	24 (66.7)	8 (25.8)
38	Repeated failure in school or at work	20 (55.6)	10 (32.3)
47	An event that seriously threatened your well-being, employment, professional status, social standing, or financial security?	19 (52.8)	2 (6.5)
43	Physical or sexual abuse	18 (50.0)	3 (9.7)
41	Being beaten up or physically threatened	17 (47.2)	9 (29.0)
48	A serious medical illness, surgery, or other distressing medical procedure	14 (38.9)	8 (25.8)
44	Rape	10 (27.8)	5 (16.1)
49	A serious accident or injury (for example, an automobile accident, or plane crash)?	6 (16.7)	5 (16.1)
52	Being a victim of a crime (for example, being robbed, assaulted, or mugged)?	6 (16.7)	4 (12.9)
45	Being the object of a lawsuit or disciplinary action	5 (13.9)	4 (12.9)
46	Being arrested or indicted for a crime?	5 (13.9)	1 (3.2)
51	Being threatened by criminals or terrorists?	4 (11.1)	1 (3.2)
54	Being imprisoned, kidnapped, tortured, or held hostage	2 (5.6)	3 (9.7)
50	A disaster (for example, a hurricane, flood, fire, tornado, earthquake, or explosion)?	1 (2.8)	4 (12.9)
53	Being in a war zone	1 (2.8)	1 (3.2)

**Table 2:** Comparison of AdAS Spectrum, TALS-SR, CAT-Q and EDI-2 scores between groups.

	LTS Mean ± SD	HTS Mean ± SD	t	*p*	Cohen’s d	C.I. 95%	
						Lower	Upper
**AdAS Spectrum**							
**Total score**	68.00 ± 24.39	80.12 ± 24.91	−1.978	0.046 *	−0.491	−0.984	−0.005
**TALS-SR**							
**Total score**	24.35 ± 19.00	63.25 ± 15.94	−12.955	0.001 **	−2.166	−2.572	−1.756
**CAT-Q**							
**Compensation**	24.03 ± 9.03	29.88 ± 13.07	−2.114	0.038 *	−0.516	−1.009	−0.019
**Masking**	30.39 ± 8.42	35.50 ± 9.30	−2.315	0.037 *	−0.575	−1.070	−0.706
**Assimilation**	27.13 ± 7.58	32.88 ± 12.72	−2.237	0.026 *	−0.543	−1.037	−0.405
**Total score**	81.55 ± 20.00	98.26 ± 31.12	−2.598	0.015 *	−0.633	−1.129	−0.131
**EDI-2**							
**Drive for thinness**	9.74 ± 6.15	12.47 ± 6.88	−1.679	0.077	−0.417	−0.907	−0.007
**Bulimia**	5.97 ± 4.87	7.18 ± 6.48	−0.843	0.387	−0.209	−0.697	0.279
**Body dissatisfaction**	9.32 ± 5.93	11.44 ± 6.48	−1.371	0.174	−0.340	−0.829	0.151
**Ineffectiveness**	8.48 ± 6.23	12.20 ± 6.16	−2.419	0.018 *	−0.601	−1.096	−0.100
**Perfectionism**	6.35 ± 5.08	7.76 ± 3.43	−1.299	0.311	−0.328	−0.817	0.163
**Interpersonal distrust**	4.26 ± 3.71	5.82 ± 4.11	−1.604	0.108	−0.398	−0.888	0.095
**Interoceptive awareness**	8.35 ± 6.15	13.26 ± 6.36	−3.156	0.004 **	−0.784	−1.287	−0.275
**Maturity fears**	7.97 ± 5.40	9.32 ± 5.27	−0.1024	0.306	−0.254	−0.742	0.235
**Asceticism**	6.45 ± 3.69	7.32 ± 3.73	−0.946	0.316	−0.235	−0.723	0.254
**Impulsivity**	8.06 ± 5.75	11.44 ± 7.16	−2.083	0.042 *	−0.517	−1.010	−0.020
**Social insecurity**	4.58 ± 4.29	7.50 ± 4.89	−2.549	0.012 *	−0.633	−1.130	−0.132
**Total score**	79.55 ± 35.41	105.73 ± 39.69	−2.796	0.004 **	−0.694	−1.193	−0.190

* significant for *p* < 0.05; ** significant for *p* < 0.01; LTS: low trauma symptoms; HTS: high trauma symptoms.

**Table 3:** Pearson’s correlation coefficients between CAT-Q and TALS-SR total scores in the overall sample.

		CAT-Q Total Score
**TALS-SR Total score**		0.246
	*p*	0.048 *
C.I. 95%	Lower	0.021
	Upper	0.448

* Significant for *p* < 0.05.

**Table 4:** Linear regression analyses with the CAT-Q total score as a dependent variable and TALS-SR total score as an independent variable in the overall group.

	B (S.E.)	Beta	t	*p*	C.I. 95%	
					Lower	Upper
**Constant**	76.006 (6.537)	-	9.710	<0.001 *	62.563	87.924
**TALS-SR total score**	0.289 (0.121)	0.246	2.018	0.014 *	0.042	0.528

R^2^: 0.061; Adjusted R^2^: 0.046; * Significant for *p* < 0.05.

**Table 5:** Linear regression analyses with the CAT-Q total score as a dependent variable and TALS-SR domains scores as independent variables in the overall group.

	B (S.E.)	Beta	T	*p*	C.I. 95%		VIF
					Lower	Upper	
**Constant**	88.342 (8.270)	-	10.590	0.001 *	72.477	106.356	-
**Loss Events**	−5.996 (2.607)	−0.462	−2.638	0.024 *	−10.402	−0.280	2.519
**Grief Reactions**	1.142 (0.844)	0.293	1.652	0.173	−0.611	2.674	2.577
**Pot.Traum.Events**	−620 (1.234)	-0.082	−0.463	0.607	−2.954	1.950	2.543
**Reaction to Losses or Upsetting Events**	−0.205 (1.477)	-0.032	−0.134	0.898	−3.233	2.713	4.697
**Re-Experiencing**	−3.136(2.096)	−0.330	−1.474	0.116	−6.723	1.421	4.108
**Avoid. & Numb.**	1.448 (1.558)	0.163	0.766	0.330	−1.794	4.425	3.734
**Maladaptive Coping**	5.904 (2.103)	0.521	2.926	0.005 *	1.614	10.023	2.601
**Arousal**	0.559 (2.851)	0.036	0.208	0.834	−6.205	5.267	2.510
**Pers. Charact./Risk Factors**	3.486 (2.367)	0.242	1.473	0.136	−1.707	7.707	2.211

R^2^: 0.330; Adjusted R^2^: 0.220; * Significant for *p* < 0.05.

## Data Availability

The original contributions presented in this study are included in the article. Further inquiries can be directed to the corresponding author.

## Abbreviations

The following abbreviations are used in this manuscript:

ED	Eating disorder
DSM-5-TR	Diagnostic and Statistical Manual for Mental Disorders, fifth edition, text revision
AN	Anorexia nervosa
BN	Bulimia nervosa
BED	Binge eating disorder
ATs	Autistic traits
PTSD	Post-traumatic stress disorder
HPA	Hypothalamic–pituitary–adrenal axis
ASD	Autism spectrum disorder
HTS	High trauma symptomatology
LTS	Low trauma symptomatology
AOUP	Azienda Ospedaliero Universitaria Pisana
AdAS Spectrum	Adult Autism Subthreshold Spectrum
TALS-SR	Trauma and Loss Spectrum—Self Report
CAT-Q	Camouflaging Autistic Traits questionnaire
EDI-2	Eating Disorder Inventory
AN-R	Anorexia nervosa—restrictive
AN-BP	Anorexia Nervosa—binge–purging
